# Digital Interventions Supporting Self-care in People With Type 2 Diabetes Across Greater Manchester (Greater Manchester Diabetes My Way): Protocol for a Mixed Methods Evaluation

**DOI:** 10.2196/26237

**Published:** 2022-08-17

**Authors:** Joanna Goldthorpe, Thomas Allen, Joanna Brooks, Evangelos Kontopantelis, Fiona Holland, Charlie Moss, Deborah J Wake, Doogie Brodie, Scott G Cunningham, Naresh Kanumilli, Hannah Bishop, Ewan Jones, Nicola Milne, Steve Ball, Mark Jenkins, Bogna Nicinska, Martina Ratto, Michael Morgan-Curran, Gemma Johnson, Martin K Rutter

**Affiliations:** 1 Manchester Centre for Health Psychology School of Health Sciences University of Manchester Manchester United Kingdom; 2 Manchester Centre for Health Economics University of Manchester Manchester United Kingdom; 3 Division of Informatics, Imaging and Data Sciences Faculty of Biology, Medicine and Health University of Manchester Manchester United Kingdom; 4 Division of Population Health, Health Services Research and Primary Care Faculty of Biology, Medicine and Health University of Manchester Manchester United Kingdom; 5 My Way Digital Health Dundee United Kingdom; 6 Centre for Medical Informatics Usher Institute University of Edinburgh Edinburgh United Kingdom; 7 Population Health & Genomics School of Medicine University of Dundee Dundee United Kingdom; 8 Northenden Group Practice Manchester United Kingdom; 9 Diabetes, Endocrinology & Metabolism Centre Manchester Royal Infirmary Manchester United Kingdom; 10 Greater Manchester & Eastern Cheshire Strategic Clinical Networks Greater Manchester Health & Social Care Partnership Manchester United Kingdom; 11 Oviva Health London United Kingdom; 12 Beingwell Group English Institute of Sport Sheffield United Kingdom; 13 MyCognition Ltd London United Kingdom; 14 Changing Health Limited Newcastle upon Tyne United Kingdom; 15 Division of Diabetes, Endocrinology & Gastroenterology School of Medical Sciences University of Manchester Manchester United Kingdom

**Keywords:** diabetes, electronic health, self-management, complex intervention

## Abstract

**Background:**

Type 2 Diabetes (T2D) is common, with a prevalence of approximately 7% of the population in the United Kingdom. The quality of T2D care is inconsistent across the United Kingdom, and Greater Manchester (GM) does not currently achieve the National Institute for Health and Care Excellence treatment targets. Barriers to delivery of care include low attendance and poor engagement with local T2D interventions, which tend to consist of programs of education delivered in traditional, face-to-face clinical settings. Thus, a flexible approach to T2D management that is accessible to people from different backgrounds and communities is needed. Diabetes My Way (DMW) is a digital platform that offers a comprehensive self-management and educational program that should be accessible to a wide range of people through mobile apps and websites. Building on evidence generated by a Scotland-wide pilot study, DMW is being rolled out and tested across GM.

**Objective:**

The overarching objectives are to assess whether DMW improves outcomes for patients with T2D in the GM area, to explore the acceptability of the DMW intervention to stakeholders, and to assess the cost-effectiveness of the intervention.

**Methods:**

A mixed methods approach will be used. We will take a census approach to recruitment in that all eligible participants in GM will be invited to participate. The primary outcomes will be intervention-related changes compared with changes observed in a matched group of controls, and the secondary outcomes will be within-person intervention-related changes. The cost-effectiveness analysis will focus on obtaining reliable estimates of how each intervention affects risk factors such as HbA1c and costs across population groups. Qualitative data will be collected via semistructured interviews and focus groups and organized using template analysis.

**Results:**

As of May 10, 2021, a total of 316 participants have been recruited for the quantitative study and have successfully enrolled. A total of 278 participants attempted to register but did not have appropriate permissions set by the general practitioners to gain access to their data. In total, 10 participants have been recruited for the qualitative study (7 practitioners and 3 patients). An extension to recruitment has been granted for the quantitative element of the research, and analysis should be complete by December 2022. Recruitment and analysis for the qualitative study should be complete by December 2021.

**Conclusions:**

The findings from this study can be used both to develop the DMW system and improve accessibility and usability in more deprived populations generally, thus improving equity in access to support for T2D self-management.

**International Registered Report Identifier (IRRID):**

DERR1-10.2196/26237

## Introduction

### Background

Type 2 diabetes (T2D) is a common long-term condition that affects 7% of the population in the United Kingdom. Compared with people without T2D, those with the condition are at higher risks for a number of complications, including myocardial infarction, stroke, renal replacement therapy, blindness, and major amputation [1,2]. Psychological comorbidities are also common, with depression affecting approximately 1 in 5 adults with T2D and diabetes-related distress affecting approximately 1 in 5 adults with T2D [3]. Self-care (eg, diet, physical activity, and medication adherence) plays a critical role in the management of T2D. Poor self-care can lead to serious diabetes-related complications. Low levels of patient knowledge about T2D contribute to poor glycemic control, and there is evidence in the United Kingdom of major variation in the level of knowledge that people with T2D have about their condition [4].

The management of people with noncommunicable long-term conditions, including T2D, presents a significant challenge to health care systems globally [1]. In the United Kingdom, routine management of T2D is primarily undertaken in primary care settings. There is considerable variation in the quality of diabetes care provided across different services and localities [5]. In Greater Manchester (GM), a mainly urban area in North West England, with a population of 2.8 million people, there are approximately 150,000 adults with T2D. Across GM, there is a poor achievement of national treatment targets for delivering T2D management. Practical and financial challenges exist in delivering interventions aimed at improving T2D knowledge and self-management skills. Current self-management and learning offer in GM is a traditional group-based *structured education* service, and its uptake is low, with attendance rates of approximately 15% [6].

Digital diabetes management systems have the potential to deliver cost-effective self-management support. Diabetes My Way (DMW) is a platform for an open access website (*My Diabetes My Way*) that has been available in Scotland since 2008 to people with diabetes and their carers. Originally, the system included various multimedia resources aimed at improving self-management; from 2010, it offered users access to their clinical data in the form of an electronic personal health record. DMW aims to improve both the outcomes and the experience of people with T2D and provide them with a single care record that is shared with their clinicians. With increased access to and control of their own data, the intention is that patients are able to share decision-making and care planning with clinicians, family, and carers. Through a partner application (MyDiabetesClinical), clinicians can access patient recorded information (with patients’ permission) and provide support for clinical decision-making, care planning, and self-management advice.

By 2020, more than 60,000 people have registered for DMW (including approximately one-third of all people with type 1 diabetes). Evaluations of the system have been encouraging, with 90% of respondents to 1 survey of users reporting that engagement with DMW helped them make better use of their consultation, improved their diabetes management and improved their condition-related knowledge [7]. However, despite positive feedback from users in the Scottish cohort, the overall uptake of DMW was low, with only 5.7% of eligible patients having registered by September 2015, steadily moving to less than 20% in 2020. There was also a disproportionately lower use of DMW among older populations, lower socioeconomic groups, and minority ethnic groups [8].

DMW has evolved based on the evidence base generated from the Scottish pilot and now incorporates an app to be used with mobile devices, which includes tailored *push* decision support, including health warnings and reminders to patients and clinicians. It is this enhanced version of DMW that, as part of the GM Diabetes My Way project, will be offered to all patients with T2D in GM from August 2019 to March 2022. DMW in GM is a collaborative led by the Greater Manchester and Eastern Cheshire Strategic Clinical Networks. Their aim is to provide a more comprehensive self-management and learning offer than is currently available to people with T2D in GM by delivering an enhanced version of DMW across the region. An additional aim is to improve the uptake of the system across all patient groups.

### Integrated Digital Interventions Offered Through the DMW Platform

#### Overview

This work is part of a wider project that aims to test offer a range of other digital supporting services and materials aimed at providing flexible access to support for a wider range of people with T2D. In addition to the DMW platform, this project will offer a range of other digital supporting services and materials through the DMW platform, which will form a package of multiple offerings [9]. The adjunct interventions will include digital support around techniques to change health behaviors (Oviva and Changing Health) and digital support, which aims to assess and improve cognitive functioning (MyCognition).

*Oviva* offers 12-week personalized, frequent, one-to-one care from a diabetes specialist dietician using behavior change techniques [10]. The app or telephone is also used to maintain regular contact with the dietician. The intervention has been evaluated in 204 people with T2D recruited from practices in London. Engagement with the *Oviva* intervention was associated with a 12.8 mmol/mol reduction in hemoglobin A_1c_ (HbA_1c_) level, a 4.3 kg reduction in body weight, and a 24% diabetes remission rate [11]. In our study, we will build on this previous work by increasing the number of participants recruited from an ethnically and socially diverse population.

C*hanging Health* offers a 12-week personalized program consisting of a National Health Services (NHS)-digital-approved and Quality Institute for Self-management Education and Training–accredited app supported by a lifestyle coach trained in behavior change techniques [12]. The educational content on the app consists of short videos, articles, and interactive activities on diet and exercise that participants can view at their convenience on their mobile phone or computer. Upon completion of the educational content, participants can book telephone appointments with their lifestyle coaches at the time of their convenience. All participants will receive 100 minutes of coaching (1×20 minutes introductory call, followed by 8×10-minute calls) across the 12 weeks. The intervention was evaluated in 41 people with T2D recruited from practices in London, and engagement with the *Changing Health* intervention was associated with a 4-mmol/mol reduction in HbA_1c_, a 1.5 kg reduction in body weight, and a 1 mmHg fall in systolic blood pressure [11]. In our study, we will build on this previous work by increasing the number of participants recruited from an ethnically and socially diverse population.

MyCognition is designed to improve cognitive performance, enhance mental resilience, and reduce the impact of stress through cognitive training exercises. The MyCognition intervention can be accessed via a mobile phone or a computer. A web-based assessment tool (taking 15 minutes to complete) provides a personal report on cognitive fitness [13]. A web-based program of personalized educational resources designed to increase cognitive performance follows this. MyCognition also provides access to a personalized game-based training application that can be used for 10-15 minutes per day is also designed to increase cognitive performance. Healthy lifestyle choices are encouraged by the application. Several studies have shown statistically significant improvements in cognitive performance using the application [14-18]. Other interventions designed to improve cognitive performance appear to improve T2D self-management in small studies [18]. In the study we plan to assess the impact of MyCognition on diabetes-related distress in a large cohort of patients with T2D.

#### Acceptability

Acceptability is an important component of the successful implementation of complex interventions. Evaluations of effectiveness can be undermined by the problems of acceptability to stakeholders (patients, carers, and health service staff). If the key protagonists involved encounter barriers to engagement with the components of an intervention, the outcomes can be affected. Assessment of acceptability to participants (patients and staff) is therefore an important part of the DMW GM evaluation that could provide important insights into usability and uptake of the intervention. This is particularly pertinent, given the findings of the study indicating low and divided uptake [7].

Acceptability has been recognized as an important feature of implementation research in the Medical Research Council guidance for the development and evaluation of complex interventions [9]. This guidance recommends that acceptability is explored both in terms of stakeholders’ engagement with the components of an intervention and (for pilot and feasibility studies) with regard to aspects of an associated research study, such as randomization of participants to a control group and completion of outcome measures [19,20] published research into acceptability of complex interventions has increased in number since this guidance was issued. This work has generally focused on barriers and facilitators to engagement [21-23], and *acceptability* has lacked a coherent definition within health services research in terms of its meaning, significance, and theoretical underpinnings. In response, a theoretical framework of acceptability (TFA) has been developed by identifying a relevant and comprehensive theoretical and empirical evidence base. Although this framework is relatively new and has not been widely used and validated, it has been developed from a robust evidence base and offers researchers a useful and unique tool for quantitatively and qualitatively assessing the acceptability of complex interventions [24]. The TFA will be used to inform qualitative data collection and analysis in this study.

### Aims and Objectives

The primary aim is to assess whether digital interventions (DMW and the adjunct interventions) improve T2D self-management across GM using quantitative methods. The primary outcomes will be intervention-related changes compared with changes observed in a matched group of people with T2D not using the interventions (controls), and the secondary outcomes will be within-person intervention-related changes.

The secondary aims are (1) to assess cost-effectiveness in comparison with other services, (2) to assess the acceptability of this package of interventions using qualitative methods, and (3) to perform a process evaluation.

### Specific Objectives

The specific objectives are listed in Textbox 1.

**Specific objectives.**

**Diabetes My Way–related objectives**

- To assess intervention-related changes in hemoglobin A_1c_ (HbA_1c_) levels, systolic blood pressure, cholesterol, smoking, and body weight or BMI (compared with controls and within-person changes).
- To assess patient uptake (proportion offered intervention who take it up), engagement (time spent on the web and content viewed), user experience (usability, knowledge, and ability to self-manage), retention (proportion of people interacting with the intervention more than once), completion (proportion of people interacting with the intervention who use it within 2 months of the end date (March 31, 2022), and health care use (clinic and hospital attendance and medication use).
- To assess how Diabetes My Way (DMW) is integrated into care pathways in primary care.

**Behavioral interventions (Oviva and Changing Health)–related objectives**

- To assess average changes in HbA_1c_ levels, systolic blood pressure, cholesterol, smoking, and body weight or BMI in participants using the intervention (compared with controls and within-person changes).
- To assess patient uptake, engagement, retention, completion, and health care use (defined for DMW above except for completion, which in the case of the behavioral interventions, will be completion of the course).

**MyCognition-related objectives**

- To assess average within-person changes in *diabetes distress scores*.
- To assess changes in cognition scores.
- To assess changes in referral rates for traditional psychological interventions (compared with controls and within-person changes).

**Health economics objectives**

- To assess the net financial costs of the intervention for the health system.
- To assess the costs to innovation partners of participating in the Test Beds program.
- To assess the net financial benefits of intervention for the health system.

**Process evaluation objectives (addressed in a brief narrative report)**

- To describe the process through which the study was designed.
- To explain if the interventions were delivered in line with original plans.
- To explain if the governance arrangements for the intervention were effective and why.
- To describe whether the partnership of National Health Services (NHS) with innovator firms worked as intended and why.
- To describe whether the innovator partnerships resulted in improved technology *pull-through*.
- To describe whether the NHS has received better products or processes as a result of collaboration, testing, or learning.
- To describe the benefits to innovation partners of being part of the Test Bed program.
- To describe whether engagement by each party to the partnership been sufficient and why.
- To describe whether changes were made during implementation to ensure effective delivery of the intervention and why.
- To describe whether there were barriers and facilitators to effective delivery (and uptake of technology or services) and how were they overcome or ensured.
- To describe any unintended consequences that needed to be managed and how was this done.
- To describe to what extent is the intervention likely to be scalable and why.

**Acceptability objectives**

- To explore acceptability of DMW and any adjunct interventions.
- To explore possible mechanisms of change.
- To explore barriers and facilitators to engagement and sustained use.
- To explore any perceived benefits or drawbacks of using DMW and adjunct interventions.
- To explore and barriers and facilitators to implementation (eg, usability, information technology issues, and skills required).

## Methods

A mixed methods approach will be used [25] using a quantitative study with a nested study using qualitative data collection and analysis.

### Ethics

Ethical approval has been granted for this work through the appropriate governing body: NHS West Midlands Black Country Research Ethics Committee reference 265621 (qualitative research) and North West—Greater Manchester South Research Ethics Committee reference 261268 (full study).

### Quantitative Study

#### Design

DMW, behavioral interventions (Oviva and Changing Health) and MyCognition will be prospective controlled cohort studies. Intervention-related changes in outcomes such as glucose levels will be assessed after adjusting for prospective changes observed in matched cohorts of patients not receiving any intervention.

#### Participants and Recruitment

We will take a census approach to recruitment in that all eligible participants in GM will be invited to participate. Identification of potential participants will take place primarily through searches of primary care practice databases to identify patients with T2D. Practice searches will be facilitated by the staff of the Greater Manchester Diabetes Clinical Research Network whenever possible. Letters from the practice, in most cases, will make initial contact with potential study participants. In some cases (according to practice preference), a text message from the practice to the patient or an email sent from the practice to the patient will be used. Posters advertising the study will be made available in practice clinics and waiting rooms.

We expect that a small number of patients with T2D will be identified through their clinical teams based in hospital outpatient clinics (most people with T2D are managed in primary care) and those attending Allied Health clinics (eg, eye screening and podiatry).

#### Inclusion and Exclusion Criteria for the Main Intervention (DMW)

The inclusion and exclusion criteria for the main intervention are presented in Textbox 2.

**Figure 1:**
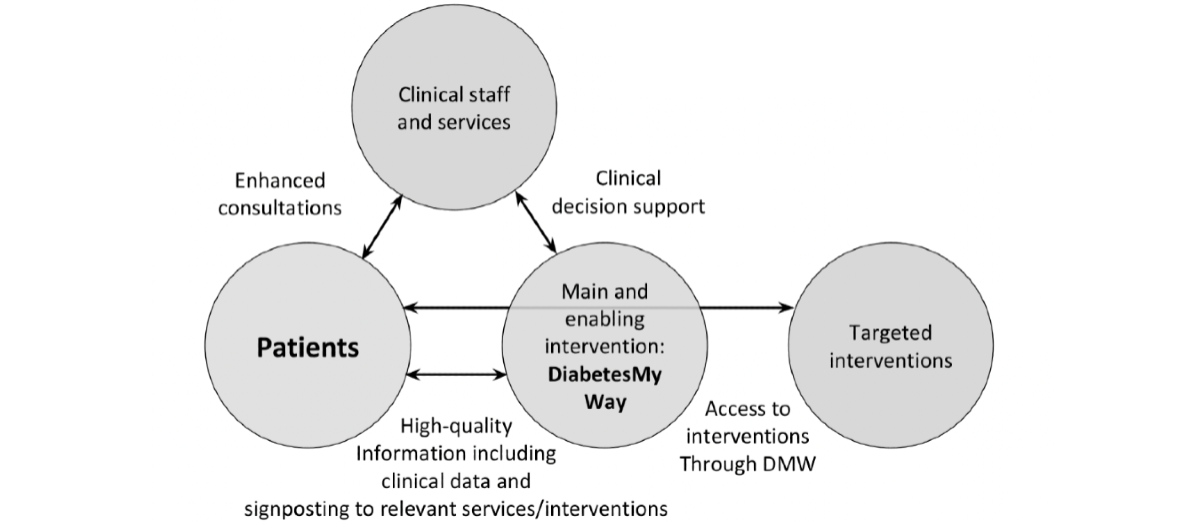
Diabetes My Way: The Main and Enabling Interventions.

The goal will be to offer DMW to all 140,000 people with T2D in GM, and a clinician-facing version will be offered to all GM primary care staff. Behavioral interventions (Oviva or Changing Health) will be offered to a total of 600 patients each. MyCognition cognitive assessment will be offered to all DMW participants, and the intervention on cognitive function will be offered to the first 600 participants. Other digital interventions will be offered to DMW participants through the DMW interface as shown in Figure 1. Participants will be offered access to *additional digital interventions* through DMW.

**Inclusion and exclusion criteria for the main intervention.**

**Inclusion criteria**

- Age ≥18 years
- Type 2 diabetes (as determined by primary or secondary care records)
- Self-certified understanding of written English
- Registered with general physician in Greater Manchester

**Exclusion criteria**

- Self-reported *severe mental illness not currently managed by a physician*.

#### Enrollment and Informed Consent

Enrollment to the DMW intervention will be facilitated through the website, and patients will be directed to the participant information sheet. Providing patients are aged ≥18 years and have a diagnosis of T2D, they will be able to express their interest in taking part in the study by submitting their contact details via the DMW website. Patients will register for web-based services via their general practitioner (GP) who will confirm their identity by checking their documentation. Patients are required to use these web-based service codes to register for DMW and answer a number of inclusion and exclusion criteria. Once registered (and where they match the criteria), the study consent form will be introduced. If and when completed and submitted, the patient’s account is activated, and they are enrolled in the study. When registered, but not matching the study criteria, the patient will be given access to DMW, but not entered into the study or consent process.

Enrollment to other digital interventions will also occur via the DMW website. On the basis of the inclusion and exclusion criteria, participants enrolled and using the DMW intervention will be invited to take part in additional substudies involving an additional digital intervention (Oviva or Changing Health allocated to alternate eligible participants or MyCognition). The above procedure for communicating participant information and obtaining informed consent will be repeated.

We will invite participants to enroll with either Oviva or Changing Health behavioral interventions until 600 have attended each intervention. These interventions will be offered to alternate participants enrolling with DMW, ensuring that the numbers receiving each intervention remain similar.

All DMW participants will be provided with the option of completing the MyCognition web-based cognitive assessment and will be offered the MyCognition intervention until 1000 have completed the intervention. When 1000 people have completed the intervention, DMW participants will continue to be offered the option of completing the MyCognition web-based cognitive assessment but will not be offered the MyCognition intervention. Participants will be invited to complete the MyCognition web-based cognitive assessment every 3 months during the study (a maximum of 3 times). Those completing these assessments, but without receiving the intervention, will act as a control group.

DMW study participants will be invited to access the other interventions through a notice shown at the top of the home dashboard page of the DMW. Opportunities to enroll will be available instantly upon successful log-in and acceptance of the study criteria.

#### Comparison Group

Within the short time frame available for the study, a randomized controlled trial will not be possible; therefore, the effectiveness of the interventions will be compared with groups of people choosing not to take up the offer of interventions. Data on this comparison group will be sourced from NHS Digital as part of the national GP Data for Planning and Research program. The comparison group will be sourced from patients registered in GM, the other inclusion and exclusion criteria being applied. Controls (not using the intervention) will be matched (up to 10:1) with study participants on age (+2 or −2 years), gender, ethnicity, and general practice. When appropriate, we will increase the number of controls per participant to enable additional matching on baseline levels of outcome measures, such as HbA_1c_ levels. Where there are clinically important differences between participants and controls in relation to other characteristics such as prevalent cardiovascular disease, we will include these variables as covariates regression models.

#### Outcomes

##### Overview

The primary outcomes will be intervention-related changes compared with changes observed in a matched group of controls, and the secondary outcomes will be within-person intervention-related changes (Textbox 3).

**Primary and secondary outcomes.**

**Diabetes My Way, Oviva, and Changing Health behavioral interventions**

- Primary outcome
  Change in hemoglobin A_1c_ levels [26,27].
- Secondary outcomes
  Changes in body weight or BMI, blood pressure, cholesterol, and smoking [28].
  Uptake, engagement, user experience, retention, completion [29], and health care use (clinic and hospital attendance and medication use).
  Impact of primary care staff on knowledge, skills, and confidence in diabetes management [30].
  Modifying effect of cognitive function [31].

**MyCognition**

- Primary outcome
  Change in diabetes distress score [32-34].
- Secondary outcomes
  Change in cognition scores.
  Changes in referral rates for traditional psychological interventions compared with controls and within-person changes.

##### User Engagement With Digital Intervention

A range of metrics will be gathered remotely with no additional burden on the participants. Measures will include the number of users offered the intervention, number of users expressing an interest in the intervention, number of active users, number of inactive users (registered but not logged onto the intervention), number of users and percentage who have viewed all learning content, number of users and of participants who have booked a coaching session (for behavioral interventions), number of coaching sessions attended, and number and percentage of participants completing digital structured education (for behavioral intervention).

### Overview of Data Items and Source of Data by Intervention in the Quantitative Study

#### Diabetes My Way

To evaluate the DMW intervention, the University of Manchester will obtain participant data from GP records via DMW, between April 1, 2016, and March 30, 2022. These data will include the following: age, sex, ethnicity, GP postcode (to assess the degree of socioeconomic deprivation) [35], diabetes type and duration, blood pressure, cholesterol, creatinine, estimated glomerular filtration rate (eGFR), smoking and body weight, height, BMI, medication, diabetes clinic attendance in primary care, and hospital visits including emergency visits.

Participant data from DMW or NHS Digital will also include ethnicity (self-provided, sometimes unreliable from primary care due to missing data), user experience or usability, service use (medication, diabetes clinic attendance in primary care, and hospital visits including emergency visits), and website activity.

Data on control participants (not using DMW) will be obtained from NHS Digital, from April 1, 2016, to March 30, 2022, and will include the following: age, sex, ethnicity, GP postcode (to assess socioeconomic deprivation), diabetes type and duration, blood pressure, cholesterol, creatinine, eGFR, smoking and body weight, height, BMI, medication, diabetes clinic attendance in primary care, and hospital visits including emergency visits.

#### Behavioral Interventions: Oviva and Changing Health

To evaluate the behavioral interventions, Oviva and Changing Health, the University of Manchester will obtain participant and control data from GP records via DMW and NHS Digital for the period April 1, 2016, to March 30, 2022, as described for DMW. Data on user experience or usability from Oviva and Changing Health participants will be transferred to the University of Manchester via DMW.

#### MyCognition

To evaluate the MyCognition intervention, the University of Manchester will obtain participant and control data from MyCognition via DMW, from GP records via DMW, and from NHS Digital for the period April 1, 2016 to March 30, 2022. Participant data transferred to the university via DMW will include cognition scores, diabetes distress scores (administered as a web-based questionnaire to participants), and usability data.

### Clinical and Biochemical Data in Study Participants

#### Overview

Clinical data collected by health care professionals during routine clinical care will enter the appropriate clinical management system (eg, GPs using the Elton Medical Information Systems health primary care electronic health record). Pseudonymization is a technique that replaces or removes all information that can be used to identify an individual. The process involves replacing names or other identifiers that are easily attributed to individuals with a study reference number. DMW will provide pseudonymized data on study participants and controls to the University of Manchester.

#### Clinical Data

The clinical data of interest includes age, sex, ethnicity, socioeconomic deprivation, diabetes type, weight, height, BMI, HbA_1c_, blood pressure level, diabetes medication, blood pressure medication, lipid lowering therapy, and service use data (attendance and nonattendance at diabetes clinics and emergency hospital visits) will be obtained from primary care records. Ethnicity will be taken from self-reported data provided at enrollment with DMW (White, South Asian, Black, mixed, or other, if *other* participants will be invited to specify using text). Socioeconomic deprivation will be assessed from the GP postcode to reduce the likelihood of participants being identified.

#### Biochemical Data

All blood sampling (eg, HbA_1c_ and serum lipids) will be conducted via routine clinical care. Participants who did not have a blood sample taken within the preceding 3-6 months will be advised through a message on the DMW website to arrange a diabetes review (weight, blood pressure, smoking status, medication review, HbA_1c_, and lipids) with their practice team at the time of enrollment in line with standard clinical care according to National Institute for Health and Care Excellence (NICE) guidance 28 [36] Participants will be advised to have repeat weight, blood pressure, medication review, and blood tests every 3-6 months in line with standard clinical care according to NICE guidance 28 [36]. Study participants will not be subject to any investigations outside of their routine care unless clinically indicated. Blood test results will be transferred to DMW from the primary care record.

#### Duration of Data Access

The University of Manchester will request primary care data on study participants for the period April 1, 2016, to March 30, 2022, through DMW This will enable a 3-year assessment of *baseline* levels of risk factors such as HbA_1c_ and blood pressure before study commencement (July 2019), and up to the end of the intervention (March 31, 2022).

### Clinical and Biochemical Data From NHS Digital in Participants and in Controls not Providing Consent

Our study involves comparing intervention-related changes in risk factors, such as HbA_1c_ levels, in study participants with the changes occurring in a control patient cohort not receiving the intervention.

NHS Digital will use their General Practice Extraction Service (GPES) to provide GP data on all patients with T2D across GM (approximately 160,000 people) between April 1, 2016, to March 31, 2022. The core data items will include age, sex, ethnicity, GP postcode (to assess socioeconomic deprivation), diabetes type and duration, blood pressure, cholesterol, creatinine, eGFR, smoking and body weight, height, BMI, medication, diabetes clinic attendance in primary care, and hospital visits including emergency visits.

To distinguish between DMW participants and nonparticipants in the GPES data, the following steps will be taken:

- DMW will supply NHS Digital with details of DMW participants including NHS numbers, GP practice with which the participant is registered, DMW generated study ID, and whether the participant is using Oviva, Changing Health, or MyCognition.
- NHS Digital will use NHS numbers of participants to create a DMW participant flag within the GPES data for GM. NHS numbers will then be removed.
- NHS Digital will supply the University of Manchester with GPES data for patients from GM with T2D.
- University of Manchester will store and analyze these data in accordance with NHS Digital requirements and Data Sharing Agreement.

Our study design requires that control participants be matched to participants receiving the intervention on age, sex, ethnicity, and socioeconomic deprivation (defined by GP postcode). Therefore, we require information on these 4 characteristics, in addition to the clinical and biochemical data. Socioeconomic deprivation level will be assessed from the GP postcode to minimize the risk of patients being identified. The University of Manchester will not receive any information that will enable DMW participants or control participants to be identified.

### Analysis

#### Overview

First, the generalizability and relevance of the intervention in the context of GM will be assessed. We will descriptively compare participants in the interventions to other populations: those who did not participate, the population of GM, and the population of England. For the comparison to those who did not take up the intervention, we will use data on age, sex, ethnicity, residence location socioeconomic deprivation quintile (2019 Index of Multiple Deprivation) and risk factors (from their electronic record) for a comparison with the participants. For the GM and England comparisons, we will compare patients based on age, sex, risk factors, and comorbidities using data sourced from NHS Digital.

Second, we will examine engagement with the intervention (as quantified by relevant metrics in the app, eg, visits and time spent), and descriptively assess how it varies across age groups, sex, ethnic groups (South Asian, Black, mixed, White, and other populations) and socioeconomic deprivation quintiles. A multiple linear regression model will be used to more formally evaluate the association between the variables listed above (age, sex, ethnicity, and socioeconomic deprivation) and engagement.

Third, we will aim to use quasi-experimental methods to evaluate the effectiveness of the intervention on each outcome by comparing prospective changes in outcomes between matched participants and controls and by performing pre- versus postintervention comparisons in participants only. Matching will be performed using the caliper method, which is a modification of the nearest neighbor matching procedure that imposes a tolerance on the difference in cohort characteristics [37]. Here, we will combine calipers relating to age range (+2 or −2 years) and with exact match of gender, ethnicity, and general practice. In the matched group approach, participants receiving the interventions will be randomly matched to nonparticipants on age, sex, ethnicity, and general practice (as a proxy for socioeconomic deprivation).

Where there are differences between participants and controls in relation to matched characteristics or other characteristics such as prevalent cardiovascular disease, we will include these variables as covariates regression models.

In the presence of time series data, we will use an interrupted time series design for each outcome of interest, which takes into account the preintervention trends of the outcome as well as the information on controls and quantify the effect of the intervention on future outcome levels. If only 2 time points are available (eg, before and after the intervention), we will use a simple linear regression model with clustered errors or a paired 2-tailed *t* test to compare means in each outcome at the 2 time points.

For example, for the main outcome, change in HbA_1c_ levels, we will compare changes from baseline (defined as April 1, 2016, to July 1, 2019) during the intervention period (July 1, 2019, to March 31, 2022) in matched participants and controls after adjusting for clinically significant differences in baseline weight, blood pressure, cholesterol, creatinine, cardiovascular disease, and smoking.

If the numbers of cases and controls are large, we will explore effect heterogeneity in subgroup analyses (as separate models or using interaction terms in the main models described above) by ethnicity, age groups, socioeconomic deprivation quintile, cognitive function (assessed by MyCognition), and level of engagement with digital interventions.

#### Missing Data

The proposed analysis allows for missing data values. We will use clinical judgment to explore possible mechanisms of missingness, and we will consider including multiple imputation in the primary analysis, irrespective of levels of missingness, assuming a missing not at random scenario is unlikely (although multiple imputation has been found to perform better than complete case analysis, in a simple missing not at random scenario as shown) [38].

#### Sample Size Calculations

##### DMW, Oviva, and Changing Health Evaluations

The primary outcome for participants and controls receiving usual care is the HbA_1c_ level after the intervention. With a ratio of 1:10 for participants to controls and assuming an SD of 15 mmol/mol in HbA_1c_ levels [39], a total of 86 participants and 430 controls provides 80% power to detect an HbA_1c_ level difference in means of 5 mmol/mol (5% significance level), which is considered the smallest clinically significant HbA_1c_ change. Assuming that 90% of participants provide data after the intervention, this requires a total of 96 participants.

Therefore, we aim to recruit at least 288 participants (96×3) to DMW from which recruitment to the 2 behavioral interventions (Oviva, n=96 and Changing Health, n=96) will occur.

If the required sample size for any intervention is not achieved, appropriate consideration will be given to the interpretation of the results in light of the large number of objectives and outcomes.

##### MyCognition Evaluation

The primary outcome is the postintervention diabetes distress score (DDS). Assuming an SD of 0.92 for DDS [34], 89 patients and 445 controls (5% significance level) provide 80% power to detect a difference in mean DDS of 0.3. Assuming 90% of participants provide data after the intervention, this requires a total of 99 participants.

### Economic Evaluation

#### Short-term Health Benefits and Consequences to Costs

In the short term, economic evaluation will focus on the cost-effectiveness of DMW measured over the intervention period (up to December 2020). Data on changes in outcomes from the quantitative evaluation, such as HbA_1c_ levels, will be compared against costs across population groups. Relevant costs will be direct NHS costs assessed from intervention-related costs and changes in routine health care use, as assessed from rates of primary care consultations and hospital attendance during the observation period. The unit costs of delivering traditional face-to-face behavior change interventions and psychological interventions are available in GM, enabling meaningful cost comparisons. Further unit costs will be sources of NHS reference costs and the unit costs of health and social care.

#### Long-term Health Benefits and Consequences to Costs

Short-term improvements in diabetes management can have longer-lasting impacts on health and costs. Expected reductions in rates of diabetes-related complications and death, by modifying cardiovascular risk factor levels, will be modeled using the United Kingdom Prospective Diabetes Study outcomes model [40]. Differences in quality-adjusted life years between DMW patients and those in the control group will be estimated.

### Qualitative Study

We will structure the interviews and analysis in relation to the TFA [24]. The TFA is a multifaceted construct that consists of seven domains:

- Affective attitude (how an individual feels about an intervention).
- Burden (the perceived amount of effort it takes to engage with an intervention).
- Ethicality (the extent to which an intervention is congruent with an individual’s belief system).
- Intervention coherence (how an individual understands the aims of an intervention and how it works).
- Opportunity costs (the extent to which an individual needs to compromise existing benefits or values to engage with the system).
- Perceived effectiveness (how well an intervention achieves the desired outcomes).
- Self-efficacy (an individual’s level of confidence that they can engage in the behaviors required to participate in an intervention).

### Participants and Setting

#### Patients

The inclusion criteria for patients are presented in Textbox 4.

We will conduct up to 20 interviews with patients currently using DMW and 10 interviews with patients choosing not to use DMW (N=30; identified by potential participant’s clinical care team). Following the provision of information relating to the supporting interventions, potential participants will be asked by members of the clinical care team for permission to pass on their contact details to the research team who will then contact the participant directly to obtain informed consent. Purposive sampling will be used to capture a sample of participants representing a range of ethnic and socioeconomic backgrounds. Participants will be offered a choice of interview settings: at the patient’s home or in a private room at either the University of Manchester, the Manchester University NHS Foundation Trust Diabetes, Endocrinology and Metabolism Centre or at a mutually agreed public location. Alternatively, participants can choose to interview over the telephone or through videoconferencing.

**Inclusion criteria for patients in the qualitative study.**

**Inclusion criteria**

- Age >18 years.
- Diagnosis of type 2 diabetes (as determined by invite to attend a specialist type 2 diabetes clinic, secondary care sites) or general practitioner records (for general practitioner practices).
- Registered with a general practitioner in Greater Manchester.
- Able to understand written English.
- No self-reported severe mental illness not currently managed by a physician.

#### Clinicians

The inclusion criteria for clinicians are presented in Textbox 5.

We will conduct up to 15 interviews with clinicians in groups stratified by ethnicity and indication of socioeconomic deprivation according to the lower super output area of the general practice or outpatient clinic. We will also aim to recruit clinicians working in a variety of roles. On the basis of conversations and meetings with the Greater Manchester Clinical Research Network, we anticipate the following key roles: (1) *general practice* (GPs or practice nurses with a specialist interest in diabetes) and (2) *hospital outpatient clinics* (consultants, specialist diabetes nurses, and health care assistants).

We will also conduct 2 focus groups, 1 with each participant group (patients and practitioners) involving 6-10 participants in each group (N=12-20). We will use the focus groups to further explore any issues of contention or special interest arising from the interviews in a peer group setting. The focus groups will take place in a private room at the University of Manchester.

All interviews and focus groups will be audio recorded and transcribed by an independent contractor using an intelligent verbatim approach. The independent contractor has been approved by the University of Manchester and is aware of guidelines relating to good clinical practice and confidentiality. We anticipate that the interviews will take between 30 and 60 minutes and focus groups will take approximately 60 minutes. Experienced interviewers with a background in psychology will conduct the interviews (JG and JB). Interviewers will attempt to make the interview conversational and informal, while remaining mindful of contextual features, such as power structures and professional boundaries. Data collection will be based on an interview schedule that allows for flexibility in pursuing topics of interest. Topic guides for the interviews have been developed based on the TFA [23] and the findings from the Scottish research [7,8]. We will develop topic guides for the focus groups based on findings from data generated by the interviews.

**Inclusion criteria for clinicians.**

**Inclusion criteria**

- Age ≥18 years.
- A registered health care practitioner working with patients diagnosed with type 2 diabetes who have been offered Diabetes My Way intervention in Greater Manchester.
- Self-certified understanding of written English.

### Recruitment to the qualitative study

#### Patients

##### Overview

Patients will be recruited from 8 GP practices and 2 hospital outpatient clinics across the GM region. When engaging with patients who meet the inclusion criteria, clinicians (GPs, practice nurses, specialist diabetes nurses, health care assistants, and consultants) will invite potential participants to provide consent for the research team to contact them to provide more information about participating in the study. If patients provide consent to contact, clinicians will give them a patient information sheet to take away. Consent to contact forms will be stored safely and securely by a named individual at each recruitment site.

##### Interviews

A member of the research team will collect consent to contact forms directly from the recruiting practices and clinics weekly and contact the patients directly using the information provided, ensuring that a minimum of 24 hours has passed since consent to contact was given. The researcher will ensure that the patient has understood the participant information, answer any questions relating to participation in the research, and if the patient agrees, will then arrange a time, date, and location for the interview to take place. When interviews have been conducted with 20 patients or data saturation reached, this process will end.

##### Focus Groups

When enough participants have been recruited to ensure data collection using interviews is complete, the process will be repeated for recruitment to the focus groups until 10 patients have taken part, at which point recruitment will cease.

#### Clinicians

##### Interviews

In total, 15 clinicians will be recruited in three different ways:

1. Via the research active clinics assisting with patient recruitment.
2. At the bimonthly GM strategic clinical network for diabetes meeting.
3. At the launch event for the DMW system.

In each of the above settings, a member of the research team will identify key clinicians who are aware of DMW and are engaging with or are likely to engage with patients who are using or have been offered the intervention. The researcher will provide the clinician with a participant information sheet and obtain consent to contact. After a minimum period of 24 hours, the researcher will contact the clinician, ensure that they have understood the participant information, answer any questions relating to the research and then arrange a time, date, and location for the interview to take place. When interviews have been conducted with 15 clinicians or data saturation is reached, this process will end, and we will begin recruitment to the focus groups.

##### Focus Groups

When enough participants have been recruited to ensure data collection using interviews is complete, the process will be repeated for recruitment to the focus groups until 10 patients have taken part, at which point recruitment will cease.

#### Informed Consent

Informed consent will be obtained from all participants by an experienced member of the research team immediately before the interview or focus group taking place, at which point participants will have the opportunity to ask further questions about the study. For face-to-face interviews and focus groups, written consent will be obtained. For telephone interviews, the researcher will read aloud the consent form and consent will be provided verbally over the telephone. This exchange will be audio recorded and later transcribed by an independent company, providing a record of the consent process.

#### Analysis

Data will be analyzed thematically using template analysis [41]. A distinct feature of template analysis is the structured development of a hierarchically organized coding template. This coding template is initially developed based on subset of data (eg, a selection of transcripts that incorporate a range of accounts—in this work, this will allow for analysis of interview data to be undertaken while data collection remains ongoing), then applied to further data and revised and refined as necessary. Codes are organized into meaningful clusters (including hierarchical relationships between codes in a cluster and lateral relationships across clusters) and a full thematic structure developed iteratively.

A further feature of template analysis is that it permits the use of *a priori* themes—themes that are identified in advance of coding as likely helpful or relevant to the analysis but which are understood as tentative and may be refined or discarded if they do not prove to be useful or appropriate. As in previous work that used the constructs of a theoretical model as *a priori* themes to facilitate initial coding [42], in this work (reflecting our particular research aims), the constructs of the TFA [23] will be drawn on as *a priori* themes to initially focus our coding.

We will use established quality-checking procedures, including critical scrutiny and constant comparison of coding. Two researchers will independently code a subset of interviews, discuss to reach consensus, and generate a first version coding template, encompassing both *a priori* and emerging themes. Once the provisional coding template is discussed and agreed, the researchers will then independently apply the coding template to further data in blocks of 5 interviews, then again meet to similarly discuss and reach full consensus. This iterative process will produce a final version template encompassing all relevant materials, which will then be applied to the full data set. At each stage of the analysis, the full research team will check the validity and consistency of coding and agree upon the final thematic framework. An audit trail will be kept, and the staged analysis process will ensure both coding and interpretation are regularly cross-checked.

### Process Evaluation

In collaboration with the digital intervention provider teams, the study team will provide a narrative report on the following observed experiences and outcomes:

- The process through which the study was designed.
- Explore whether the interventions were delivered in line with original plans.
- Explore whether governance arrangements for the intervention were effective and why.
- Explore whether the partnership of NHS with innovator firms worked as intended and why.
- Explore whether the innovator partnerships resulted in improved technology use.
- Explore whether the NHS has received better products or processes as a result of collaboration, testing, or learning.
- Explore any benefits to innovation partners of being part of the Test Bed program.
- Explore whether engagement by each party to the partnership been sufficient and why.
- Describe any changes made during implementation to ensure effective delivery of the intervention and why these were made.
- Explore barriers and facilitators to effective delivery (and uptake of technology or services) and how were they overcome or ensured.
- Report any unintended consequences that needed to be managed and how this was done.
- Explore scalability of the intervention.

### Patient and Public Involvement

DMW has had patients involved in every stage of the design, prototyping, development, implementation, and review phases of the intervention. The company receives regular feedback from patients via email, secure messaging, and web-based surveys to ensure that the intervention is genuinely patient-centered and holds regular steering group meetings, including representation by patients. In addition, all new product development work involves users. This is usually conducted through design workshops and user prototype testing in the field. Early feedback (and ongoing feedback on rollout) continues to drive changes in the DMW product range. Furthermore, our partner services engage in ongoing dialogue with its users. A person living with diabetes will be enrolled in the study steering group.

*Oviva* Diabetes Support conducts patient and public involvement through its continuous patient feedback via surveys, which is reviewed monthly, and changes to the intervention are made as appropriate.

*Changing Health* sends out feedback surveys to all users at baseline and subsequently every 3 months. The data from these surveys are used to identify potential issues and to continually refine their programs. In addition to this formal channel, *Changing Health* also has a presence on social media (Facebook and Twitter) through which they share user stories and original content of interest to the public.

### Dissemination

We will take the following steps to ensure that results are useful to the NHS and the global clinical community: *ensure effective dissemination of results, such as* (1) deliver conference presentations; (2) peer-review publications; (3) run an engagement workshop on *digital solutions to improve diabetes self-management,* which will involve senior clinicians, NHS England, public health staff, diabetes charities, academics, patients, and the general public; (4) social media postings; and (5) give radio and television interviews and p*resent the results in ways that create maximum utility for clinical users (*eg, clearly describe the practical steps necessary to introduce the system). Participants will be provided with a summary of the main study findings through the DMW website.

## Results

As of May 10, 2021, a total of 316 participants have been recruited for the quantitative study and have successfully enrolled. A total of 278 participants attempted to register but did not have appropriate permissions set by the GPs to gain access to their data. A total of 10 participants have been recruited for the qualitative study (7 practitioners and 3 patients). An extension to recruitment has been granted for the quantitative element of the research, and analysis should be complete by June 2022. Recruitment and analysis for the qualitative study should be complete by June 2021.

## Discussion

### Principal Findings

The GM DMW project seeks to implement innovative solutions to clinical and system-wide challenges in learning and self-management in T2D. The overall aim of the intervention is to deliver optimized clinical care, and the electronic provision of patients’ medical information to patients and practitioners in a timely and accessible way should contribute to achieving this aim. Patients currently struggle to effectively self-manage, and clinicians are limited by not having all relevant information in a unified care record. Furthermore, there is major variation in the level of knowledge that patients have about their diabetes, major variation in the quality of diabetes care across general practices in GM, and major practical and financial challenges in delivering behavioral interventions that support healthier lifestyles and major blocks in clinical care because of diabetes-related psychological distress. These factors are all compounded by low clinic and structured education attendance rates in some patient groups.

There is a clear need to improve patients’ ability to self-manage T2D, and the DMW electronic platform offers an accessible alternative to the current, mostly unsuccessful method of traditional classroom-style education. DMW has been proven popular with patients and practitioners in Scotland; however, uptake across Scotland has been ≈13% (in 2020), with disproportionately lower use in older populations, lower socioeconomic groups, and minority ethnic groups and risks perpetuating or augmenting health inequalities [8]. Exploring issues around the acceptability of the implementation of DMW in GM with both patients and practitioners could offer important insights into possible reasons for the lack of engagement and uptake in certain populations. The findings from this study can be used both to further develop and enhance the DMW system and to improve accessibility and usability in more deprived populations, thus improving equity in access to support for T2D self-management.

If the evaluation demonstrates that DMW and the linked digital behavioral interventions (Oviva and Changing Health) can improve self-management and risk factor levels in people with T2D, then this could lead to a step-change in diabetes management across the NHS and wider. If the economic analysis shows cost savings compared with traditional care, then this would lead to global changes in health care delivery. Therefore, the clinical, psychological, social, economic, and health care resource benefits observed through this application could have a global reach. Furthermore, other adjunct offerings could impact clinical outcomes. If MyCognition improves psychological health in people with T2D, then these data could be used to promote this approach being rolled out across the NHS, which could reduce the huge public health burden of psychological illness associated with T2D.

### Strengths and Limitations

The main strength of the qualitative study is that we will draw on the experience of a wide range of stakeholders with the aim of capturing the most diverse possible range of perspectives to inform the development of DMW and improve the overall number of patients choosing to engage with the intervention.

One of the limitations of the study is that we will not be recruiting patients who do not understand written English. This may result in a biased patient sample, which is not representative of the diverse population of GM, where an estimated 200 different languages are spoken. However, this may provide a topic for future research. Another limitation is that the study will not formally evaluate the influence of digital exclusion on study outcomes. We anticipate differences in digital literacy and the factors that influence this between participants and controls. However, our matching process, followed by multivariable regression that controls for clinically important covariates not included in the matching process, may help to reduce the influence of digital exclusion. We expect that the results of the qualitative work may provide further insights into the role that digital exclusion might have in the results.

### Conclusions

Exploring the acceptability of DMW will provide valuable insight into how stakeholders engage with the intervention and how to improve implementation and uptake within diverse populations. It will compliment a larger body of quantitative research on efficacy, generated by the wider GM Test Bed study and the mixed methods Scottish research [7,8]. By using the TFA [23], an evidence-based framework for researching the acceptability of complex interventions, we will also add to a wider body of evidence around the utility of this tool and increase the transparency and replicability of our findings.

If the use of DMW by patients with diabetes has major clinical, societal, and economic benefits, this will be a global stimulus for research in this area. Researchers and health care managers will be interested in the context in which the interventions were delivered (patients and health care system) and the methods of implementation adopted in the Test Bed. The research will have implications for pharmaceutical companies and other companies involved in producing treatments, interventions, and digital interventions in diabetes care.

This study has multiple potential impacts. In 2011, diabetes consumed 10% (£10 billion; US $13.25 billion) of the NHS budget, and when indirect costs were included (mortality data, sickness data, loss of productivity, and informal care), the cost was estimated at £23 billion (US $30.47 billion). If digital interventions have only a small impact on the self-management of T2D and its complications, then the absolute economic benefits may still be large. The largest cost of managing diabetes comes from the cost of managing its complications. If interventions targeting the management of cardiovascular risk (DMW and behavioral interventions primarily) are successful, then this could have major financial benefits in the United Kingdom and globally.

The treatment of T2D is central to the government’s 2011 National Service Framework (NSF) for diabetes. Our research addresses key NSF standards. The NSF standard 4 states the following:

All adults with diabetes will receive high-quality care...to optimise the control of their blood glucose, blood pressure and other risk factors for developing the complications of diabetes.

If our research shows the expected outcomes, then we will work with leading figures in the Department of Health, Public Health England, NHS England, and NICE to ensure that future policies and guidance include appropriate reference to our work. This project also maps to the aims of (1) the NHS 10-year plan (2019), (2) the NHS England Digital Health Strategy, (3) the National Information Board Personalised Health and Care 2020 plan, and (4) the *State of the Nation* report (2016) produced by Diabetes UK.

Digital interventions have the potential to transform the self-management of T2D and deliver major clinical, psychological, and economic benefits. This Test Bed project aims to assess the impact of a range of digital interventions delivered through DMW in an ethnically and socially diverse group of people with T2D across GM.

### Note on the COVID-19 Pandemic

The plans in relation to the study participants have not been modified because of the COVID-19 pandemic. We expected digital interventions to be effective in improving self-care. However, in the context of the COVID-19 pandemic, it is clear that digital interventions could be even more valuable to people with diabetes because access to face-to-face primary care interventions for people with T2D has been extremely limited and may remain so for many months.

Before the pandemic, the study team was in contact with NHS Digital to obtain data on control participants through the Data Access Request Service. The COVID-19 pandemic provided an alternative route to obtaining these data from NHS Digital through a notice under Regulation 3 (4) of the National Health Service (Control of Patient Information Regulations) 2002. This regulation requires NHS Digital to share confidential patient information with organizations entitled to process this under Control of Patient Information Regulations for COVID-19 purposes.

## Abbreviations

DMW: Diabetes My Way
eGFR: estimated glomerular filtration rate
GM: Greater Manchester
GP: general practitioner
GPES: General Practice Extraction Service
HbA_1c_: hemoglobin A_1c_
NHS: National Health Services
NICE: National Institute for Health and Care Excellence
NSF: National Service Framework
T2D: type 2 diabetes
TFA: theoretical framework of acceptability
